# Adeno-associated virus-mediated expression of human butyrylcholinesterase to treat organophosphate poisoning

**DOI:** 10.1371/journal.pone.0225188

**Published:** 2019-11-25

**Authors:** Vibhor Gupta, C. Linn Cadieux, Deirdre McMenamin, C. Angelica Medina-Jaszek, Muhammad Arif, Omua Ahonkhai, Erik Wielechowski, Maryam Taheri, Yan Che, Tamara Goode, Maria P. Limberis, Mingyao Li, Douglas M. Cerasoli, Anna P. Tretiakova, James M. Wilson

**Affiliations:** 1 Gene Therapy Program, Department of Medicine, Perelman School of Medicine, University of Pennsylvania, Philadelphia, Pennsylvania, United States of America; 2 United States Army Medical Research Institute of Chemical Defense, Maryland, United States of America; 3 Department of Biostatistics and Epidemiology, Perelman School of Medicine, University of Pennsylvania, Philadelphia, Pennsylvania, United States of America; Weizmann Institute of Science, ISRAEL

## Abstract

Rare diseases defined by genetic mutations are classic targets for gene therapy. More recently, researchers expanded the use of gene therapy in non-clinical studies to infectious diseases through the delivery of vectorized antibodies to well-defined antigens. Here, we further extend the utility of gene therapy beyond the “accepted” indications to include organophosphate poisoning. There are no approved preventives for the multi-organ damage resulting from acute or chronic exposure to organophosphates. We show that a single intramuscular injection of adeno-associated virus vector produces peak expression (~0.5 mg/ml) of active human butyrylcholinesterase (hBChE) in mice serum within 3–4 weeks post-treatment. This expression is sustained for up to 140 days post-injection with no silencing. Sustained expression of hBChE provided dose-dependent protection against VX in male and female mice despite detectable antibodies to hBChE in some mice, thereby demonstrating that expression of hBChE *in vivo* in mouse muscle is an effective prophylactic against organophosphate poisoning.

## Introduction

Organophosphate (OP) compounds constitute a major public health risk, as demonstrated by multiple casualties during terror attacks [1, 2]. Additionally, long-term chronic exposure to OPs such as in agricultural work, has been shown to impact executive function and memory [3]. OPs act by inhibiting acetylcholinesterase (AChE) in an irreversible manner [4], and acute high-dose exposures may lead to convulsions, respiratory arrest, brain damage, and death. Current post-exposure treatments rely on muscarinic receptor antagonists (atropine), anticonvulsants (diazepam), or AChE reactivators (pralidoxime), which improve survival but do not protect from brain damage [5]. The stoichiometric scavenger human butyrylcholinesterase (hBChE) can inactivate OPs before they bind to AChE, thereby preventing brain damage [6]. However, the practical utility of plasma-purified hBChE is limited, particularly in a prophylactic setting, due to the need for frequent intravenous infusions and the lack of manufacturing scalability. Furthermore, *in vitro* [7]- and *in vivo* [8, 9]-produced recombinant hBChE [10] exhibit suboptimal pharmacokinetics. Vectorization of hBChE might overcome these challenges. Adenovirus (Ad)-mediated delivery of hBChE into mouse liver has shown efficacy, albeit with a narrow protection window of a few days due to Ad immunogenicity [11–13]. An alternative strategy for vectorizing hBChE is to use adeno-associated virus (AAV) vectors, which have established safety and efficacy profiles in humans [14].

Here, we evaluated an AAV-BChE vector as a prophylactic for OP poisoning. We used a bi-cistronic vector design to co-express hBChE and polyproline peptides, derived from PRIMA1 or lamellipodin. This design facilitated the formation of fully active tetrameric hBChE [15, 16]. Studies previously demonstrated that intravenous injection of recombinant AAV vectors delivering modified hBChE into liver results in sustained protein expression for over a year [17]. Normally, human muscle expresses low levels of hBChE [18], making it unclear whether muscle is capable of expressing high levels of functional hBChE. For the first time, we demonstrate that AAV-transduced muscle supports the long-term systemic expression of efficacious levels of fully functional hBChE.

## Materials and methods

### Animal studies

C57BL/6J RAG knockout (KO) (B6.129S7-Rag1^tm1mom/^J Jackson Labs, Stock no. 002216) and BChE KO male mice (B6.129S1-Bchetm1Loc/J Jackson Labs, Stock no. 008087), 6–8 weeks old, were sourced from the Jackson laboratory. All mice were monitored daily and housed under controlled conditions in the barrier of the animal facility of the Translational Research Laboratories at the University of Pennsylvania. Experimental protocols were approved by and performed according to the guidelines of the Institutional Animal Care and Use Committee of the University of Pennsylvania and approved by the ACURO. Each vector was quantified by quantitative or digital PCR and diluted in sterile phosphate-buffered saline (PBS) to the required dose. Prior to vector administration, mice were anesthetized by intraperitoneal injection of a mixture of 70 mg/kg ketamine and 7 mg/kg xylazine. The diluted vector was then injected aseptically into the left gastrocnemius muscle. Bleeds were collected from the retro-orbital sinus to analyze transgene expression in serum. For the butyrylcholine challenge study, 500 mg/kg butyrylcholine iodide (Sigma Aldrich; 20780-25G-F) was given intraperitoneally in a 250 μl volume. Mice were observed under veterinary care for 30 minutes. Mice that showed severe signs of poisoning (tremors, seizures, twitching) were euthanized by asphyxiation with carbon dioxide, followed by cervical dislocation.

For VX challenge studies (performed at the USAMRICD facility), 6–8 week-old male and female ES1 KO mice (B6(Cg)-Ces1ctm1.1Loc/J, Jackson Labs, stock number: 014096) in designated groups were given AAV-BChE vectors as described above and later challenged by providing a single subcutaneous dose of 2×LD_50_ VX. Mice were monitored for 1 hour following the challenge and periodically thereafter. Blood from ES1 KO mice was collected via tail nick and plasma was isolated for hBChE expression and an activity assay.

The experimental protocol was approved by the Animal Care and Use Committee at the United States Army Medical Research Institute of Chemical Defense, and all procedures were conducted in accordance with the principles stated in the Guide for the Care and Use of Laboratory Animals and the Animal Welfare Act of 1966 (P.L. 89–544), as amended.

### Vector construction and viral production

All AAV vectors were produced by the Penn Vector Core at the University of Pennsylvania as described previously [19]. In order to optimize the expression of hBChE, different ORFs were designed with various combination of promoters (CMV, UbC, CB7), leader sequences (hIL2, innate), and proline-rich peptides (PRIMA1, LPDN, or no peptide) in the presence or absence of a 5’UTR (c-myc) and 3’UTR (WPRE); amino acid and coding sequences are displayed in the S1 sequence listings. Further, eight codon variations were used to develop the most stable and active form of hBChE. For expression *in vivo*, different constructs were further packaged in the AAV8 capsid and titered by the Vector Core facility at Gene Therapy Program at the University of Pennsylvania.

### BChE expression and activity assays

BChE expression in serially diluted mouse serum or plasma was quantified using a commercially available hBChE Quantikine ELISA kit from R&D Systems (catalog number: DBCHE0). We followed the protocol recommended by the manufacturer for assay performance and analysis. hBChE activity was assayed using modified Ellman’s method [20].

### In-gel activity assay and Western blot

**S**erum samples (1.5 μl) diluted in PBS were loaded and resolved in Novex WedgeWell 4–20% Tris-Glycine gel for 2 hours at 150 Volts at 4°C. The gel was rinsed with 0.1 M phosphate buffer pH 7.6. For the butyrylcholinesterase in-gel activity assay, the gel was stained with 2 mM butyrylthiocholine using the Roots-Karnovsky staining method [21]. For immunoblotting, the native gel was transferred to a polyvinylidene difluoride membrane using Trans-blot Turbo from Bio-Rad. The membrane was blocked for 1 hour at room temperature using Odyssey^®^ blocking buffer. The membrane was incubated overnight with Novus; NBP1-85633 Rabbit polyclonal anti-BChE antibody (1: 3,000) at 4°C in PBS+0.05% Tween-20. The membrane was further washed with PBS + 0.05% Tween-20 and incubated with a IRDye^®^ 800CW Goat anti-rabbit (1: 10,000) antibody in blocking buffer at room temperature for 1 hour. Additional washes were performed, and the blot was imaged using an Odyssey^®^ CLx imaging instrument.

### Statistical analysis

The expression profile of hBChE was analyzed using a Wilcoxon rank-sum test with the function “wilcox.test” within the R Program (version 3.4.2; https://cran.r-project.org). Comparison of survival rates was performed using a one-sided Fisher exact test within the R program using the function “fisher.test”. Statistical significance was set at the 0.05 level. hBChE (accession number P06276) expression (ng/ml) and activity (units/ml) is presented as the mean of all mice/group ± SD.

## Results and discussion

**Fig 1:**
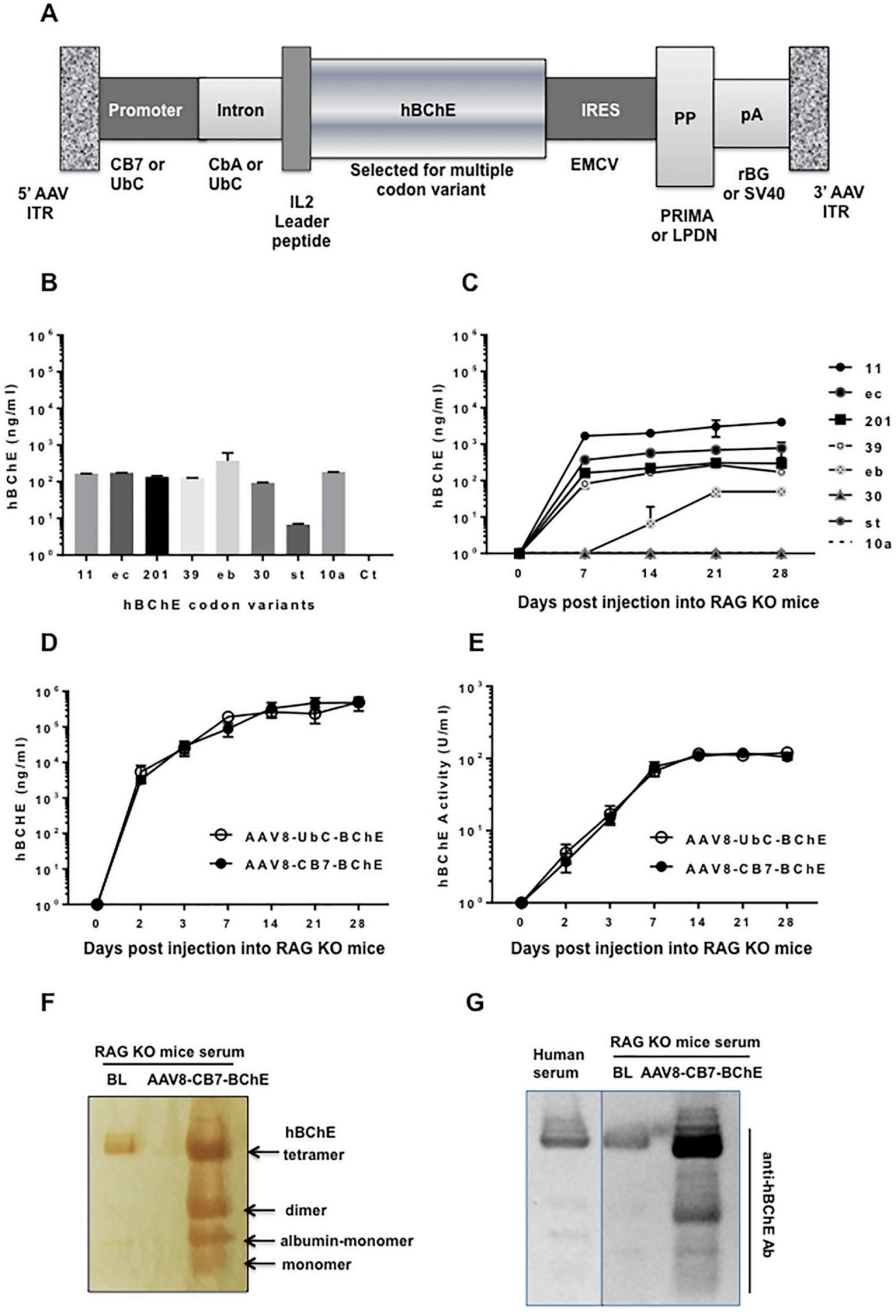
Optimizing AAV-mediated hBChE expression in serum. **(A)** Schematic representation of the AAV vector construct and the variables used to optimize maximal hBChE expression. **(B)** *In vitro* expression (ng/ml) of hBChE in the supernatant of HEK293 cells transfected with plasmids expressing different codon-optimized hBChE genes under the control of the CMV promoter including un-transfected control (Ct). **(C)** *In vivo* expression (ng/ml) of hBChE in the serum of RAG KO male mice (n = 5/group) injected IM with 10^11^ GC/mouse of an AAV vector expressing different codon-optimized hBChE genes expressed under the CMV promoter. **(D)** Expression (ng/ml) and **(E)** activity (units/ml) of hBChE protein in serum of RAG KO male mice (n = 5/group) injected IM with 10^11^ GC/mouse of AAV8-UbC-BChE or AAV8-CB7-BChE vector. **(F)** In-gel activity assay and **(G)** native Western blot of hBChE expressed by AAV8-CB7-BChE vector in RAG KO mice serum. Data represents the average of all mice (n) ± SD. BL = Baseline.

hBChE, sometimes called pseudocholinesterase or serum cholinesterase, is produced by liver and is secreted into the bloodstream. hBChE is also detectable at neuromuscular junctions, in glial cells, and in axons [22]. hBChE degrades choline esters, acts as a stoichiometric scavenger for OPs, and is irreversibly inhibited when initial phosphorylation of the active site serine residue by the OP is followed by enzyme aging [4]. The normal serum concentration of hBChE is 2–5 μg/ml [22, 23]. In order to achieve efficacious expression of a pharmacologically active protein, we optimized various vector elements across the gene expression cassette (Fig 1A), including a number of hBChE gene-coding sequences to achieve optimal protein expression *in vitro* (Fig 1B) and *in vivo* (Fig 1C). Our results show that *in vitro* expression of hBChE does not track with *in vivo* expression, emphasizing the importance of *in vivo* studies for vector optimization.

The optimal constructs were AAV8.UbC.hIL2.BChE11.IRES.PRIMA1.SV40 (AAV8-UbC-BChE) and AAV8.CB7.CI.hIL2.BChE11.IRES.LPDN.rBG (AAV8-CB7-BChE). Initial *in vivo* studies were performed in RAG KO mice to avoid expression data being confounded by immunology. In RAG KO mice, each vector produced sustained expression of ~0.5 mg/ml of active hBChE in serum at a single intramuscular (IM) dose of 10^11^ genome copies (GC)/mouse (Fig 1D and 1E). Long-term hBChE expression was similar in immunocompetent (BChE KO) mice (S1 Fig). Previous studies have reported similar sustained expression (up to 16 months) after intravenous administration of AAV-BCHE vectors into BCHE KO mice [17]. Additionally, while we did detect IgG antibodies capable of binding BChE in some ES1 KO mice (S2 Fig), those antibodies appeared to be non-neutralizing. AAV is a preferred way to deliver secreted proteins to an animal due to tolerization effects, as compared to direct injections of recombinant protein [24–26]. Although it is known that injection of purified hBChE into BChE KO mice results in immunization [27], it is not surprising that antibody responses to hBChE were not as strong as responses to hBChE delivered by recombinant AAV vector. An in-gel activity assay (Fig 1F; S3 Fig) and a native Western blot (Fig 1G; S4 Fig) showed the expected functionality and native tetrameric structure of AAV-delivered hBChE protein in serum from treated mice.

**Abbreviations:** AAV8 = adeno-associated virus capsid type 8; AAV8-UbC-BChE = AAV8.UbC.hIL2.BuChE11.IRES.PRIMA1.SV40; AAV8-CB7-BChE = AAV8.CB7.CI.IL2.BuChE11.IRES.LPDN.rB CB7 = chicken β-actin promoter; CbA = chicken β-actin; EMCV = encephalomyocarditis virus IRES; hBChE = human butyrylcholinesterase; IL2 = interleukin leader peptide 2; IRES = internal ribosome entry site; ITR = inverted terminal repeats; LPDN = lamellipodin; pp = polyproline rich peptide; PRIMA = proline-rich membrane anchor; rBG = rabbit β-globin polyadenylation sequence; SV40 = simian virus 40 polyadenylation sequence; UbC = ubiquitin C promoter.

**Fig 2:**
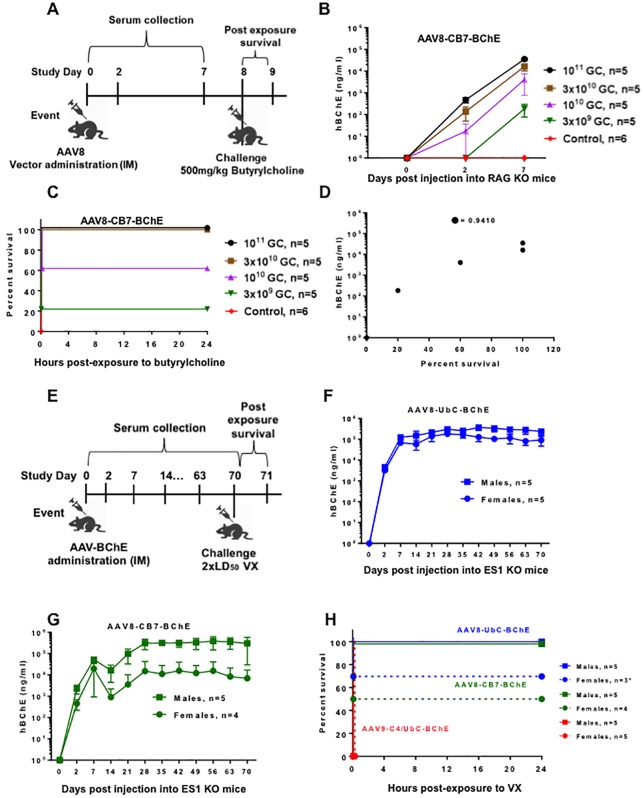
Efficacy of AAV-mediated hBChE against lethal challenge in mice. **(A)** Butyrylcholine challenge study design in male BChE KO mice (n = 5/group) injected IM with AAV8-CB7-BChE at different doses (GC/mouse) and untreated control mice (n = 6). At study day 0, 2, and 7, **(B)** hBChE expression (ng/ml) in serum was analyzed. **(C)** Survival was determined after challenge with 500 mg/kg of butyrylcholine on day 8 post-vector injection. **(D)** Correlation plot between hBChE expression and percent survival. **(E)** VX challenge study design in ES1 KO mice (n = 10/ group) injected IM with three AAV vectors expressing hBChE individually. At study day 0, 2, 7 and weekly thereafter, hBChE expression (ng/ml) was measured in plasma of mice injected IM with **(F)** AAV8-UbC-BChE, **(G)** AAV8-CB7-BChE, or AAV9-C4/UbC-BChE (S3 Fig) at 10^12^ GC/mouse. **(H)** Survival was observed after challenge with 2xLD_50_ VX at day 70 post-vector administration. Data represents the average of all mice (n) ± SD. *Two mice were excluded from the survival analysis due to inaccurate VX dosage.

To investigate the efficacy of the AAV-BChE vectors in a surrogate model, we injected five groups of BChE-KO male mice (n = 5/group) IM with doses of AAV8-CB7-BChE vector ranging from 3x10^9^ to 10^11^ GC/mouse; group 6 (n = 6) served as a naïve control (Fig 2A). We confirmed hBChE expression in serum (Fig 2B). On day eight post-vector injection, we challenged all groups with 500 mg/kg butyrylcholine administered via intraperitoneal injection [28]. All control mice showed signs of severe poisoning (tremors, seizures, and twitching) and were euthanized within minutes of exposure. By contrast, mice treated with AAV8-CB7-BChE showed a survival rate that correlated with vector dose; a minimum effective dose of 3x10^10^ GC/mouse enabled 100% survival following exposure to the agent (Fig 2C). We observed a significant correlation between the proportion of survival and BChE expression level on a log scale with a Pearson correlation of 0.941 (Fig 2D). The *p* value for testing co-relation is 0.0170. The survival rate of two top-dosed animals (10^11^ and 3x10^10^) with 100% survival in net 24 hr post-challenge was not significant (*p* = 0.057) as compared to the control group with 100% mortality; however, the animal number per group is only 5.

Rodents express high levels of endogenous carboxylesterase (ES1) in blood, which non-specifically neutralizes a significant amount of OPs during challenge studies. To abolish this native activity and rigorously evaluate the protection afforded by AAV-BChE, we used ES1 KO mice [29] for nerve agent challenge studies. We divided ES1 KO mice into three groups (5 males and 5 females per group) and injected them IM with three AAV-BChE vectors at 10^12^ GC/mouse (Fig 2E). Group 1 and 2 received the two highest hBChE-expressing vectors (AAV8-UbC-BChE and AAV8-CB7.BChE, respectively); group 3 received a low hBChE-expressing vector (AAV9-C4/UbC-BChE). We analyzed hBChE expression in plasma (Fig 2F and 2G, and S5 Fig). At study day 70, we challenged mice with 2×LD_50_ O-ethyl S-[2-(diisopropylamino)ethyl]methylphosphonothioate (VX).

VX is one of the most notorious and toxic synthetic chemical agents of the OP class developed as a chemical warfare agent. All mice (n = 10) injected IM with the low hBChE-expressing vector (AAV9-C4/UbC-BChE) died within a mean 15 minutes of exposure (Fig 2H) due to suboptimal levels of hBChE in plasma (S5 Fig). The survival rate was significantly greater for mice injected with high hBChE-expressing vectors (AAV8-UbC-BChE, p = 0.01; AAV8-CB7-BChE, p = 0.001) (Fig 2H). We compared survival rates between vector groups using a one-sided Fisher exact test within the R program using the function “fisher.test”. The analysis was stratified by gender and combined. We assessed statistical significance at the 0.05 level. As compared to the AAV-C4/UbC vector, survival of animals (male and female combined) treated with the other two vectors AAV8-UbC-BChE (p = 0.009) and AAV8-CB7-BChE (p = 0.001) was significantly higher. Male mice injected with either of the two highest expressing vectors experienced no mortality; however, we observed 35–50% mortality in female mice, correlating with lower hBChE expression (Fig 2F and 2G). Approximately 2.2 μM of circulating hBChE is required to neutralize 2×LD_50_ VX. We achieved this level of expression within three to four weeks in male mice (n = 5) using either of the two top expression vectors (AAV8-UbC-BChE and AAV8-CB7-BChE, respectively) at an IM dose of 10^12^ GC/mouse (S6 Fig).

**Expression Threshold:** A total of 13 out of 29 animals did not survive the challenge; data show **mean+3SD** hBChE expression level (at day 70) of **5.0X10**^**4**^ **ng/ml**.

**Abbreviations:** AAV8 = adeno associated virus capsid type 8; AAV8-UbC-BChE = AAV8.UbC.hIL2.BuChE11.IRES.PRIMA1.SV40; AAV8-CB7-BChE = AAV8.CB7.CI.IL2.BuChE11.IRES.LPDN.rBG, AAV9-C4/UbC-BChE = AAV9.C4/UbC.hIL2.BuChE11.IRES.LPDN.rBG; CB7 = chicken β-actin promoter; hBChE = human butyrylcholinesterase; UbC = ubiquitin C promoter;

Our results show that AAV-BChE provides sustained expression of active hBChE after injection into the muscle and protects the host from acute exposure to OP, and that the efficiency of this protection correlates with hBChE levels. Because of dose-dependent stochiometric protection, vector dose can be adjusted to achieve protection at higher exposure levels. While the translatability of intramuscular AAV-mediated delivery of secreted proteins from non-human primates to humans has not yet been established, in our experience there is a good agreement when similar doses (GC/kg) are administered to mice and non-human primates [30, 31]. These data highlight the prophylactic efficacy of AAV-BChE gene therapy against exposure to OPs and uncover its potential as a treatment for other serious medical conditions like prolonged apnea in hBChE-deficient patients during anesthesia [32].

## Supporting information

S1 Text hBChE vector construct sequences. (DOCX)

S1 File Abbreviations. (DOCX)

S1 Fig Long-term expression of hBChE (ng/ml) in the serum. Male BChE KO mice (n = 3/group) were injected IM with 10^11^ GC/mouse of AAV-BChE vectors as shown. Data is shown as the average of all mice (n) ± SD.
(DOCX)

S2 Fig Expression of anti-hBChE IgG antibodies in ES1 KO mice. Male (blue squares) and female (red circles) ES1 KO mice (n = 5/group) were injected IM with 10^12^ GC/mouse of AAV9-C4/UbC-BChE or AAV9-CB7-BChE vectors as shown. Data from an individual animal is shown. Brief methodology: Purified hBChE protein (1μg/ml, diluted in PBS) was used to coat high binding 96-well plates overnight at 4°C. The next day, plates were washed 3X with PBST and blocked with 1% BSA in PBS. Later on, 50 μl of different serum dilutions were added to all wells and incubated for 1 hr at room temperature. Plates were then washed 3X with PBST, and treated with primary antibodies (anti-mouse IgG-Biotin, 0.2ug/ml, catalogue# ab6788, 3 μl in 30 ml PBST) diluted in PBST as per the manufacturer’s recommendation; for 1 hr at room temperature. Plates were washed again, treated with secondary antibody (Biotin-HRP) and developed for reading optical density (OD) at 450 nm.
(DOCX)

S3 Fig Raw data for in-gel activity assay for Fig 1F. In-gel activity assay of hBChE expressed by AAV8-CB7-BChE vector in RAG KO mice serum. BL = Baseline.
(DOCX)

S4 Fig Raw data for Western blot for Fig 1G. Raw data for Western blot of hBChE expressed by AAV8-CB7-BChE vector in RAG KO mice serum.
(DOCX)

S5 Fig Expression of hBChE (ng/ml) in the plasma of ES1 KO mice. Male and female ES1 KO mice (n = 5/group) were injected IM with 10^12^ GC/mouse of AAV9-C4/UbC-BChE vector as shown. Data is shown as the average of all mice (n) ± SD. The post-challenge survival is shown in the main article (Fig 2H).
(DOCX)

S6 Fig Expression of hBChE (μM) in the plasma of ES1 KO mice. Male and female ES1 KO mice (n = 5/group) were injected IM with 10^12^ GC/mouse of different AAV-BChE vectors as shown. Approximately 2.2 μM of hBChE is required to neutralize 2×LD50 VX. Data is shown as the average of all mice (n) ± SD.
(DOCX)
