# Supplementary material for: Adeno-associated virus-mediated expression of human butyrylcholinesterase to treat organophosphate poisoning
Source: PLoS One. 2019 Nov 25;14(11):e0225188. doi: 10.1371/journal.pone.0225188 (PMC6876934; doi:10.1371/journal.pone.0225188)
Supplement: S1 Text — (DOCX) [file pone.0225188.s001.docx]

**Text S1. hBChE vector construct sequences**

>BCHEst

atgagaatgcaattattattattaattgcattatcattagcattagttacaaattcagaagatgatattattattgcaacaaaaaatggtaaagttagaggtatgaatttaacagtttttggtggtacagttacagcatttttaggtattccatatgcacaaccaccattaggtagattaagatttaaaaaaccacaatcattaacaaaatggtcagatatttggaatgcaacaaaatatgcaaattcatgttgtcaaaatattgatcaatcatttccaggttttcatggttcagaaatgtggaatccaaatacagatttatcagaagattgtttatatttaaatgtttggattccagcaccaaaaccaaaaaatgcaacagttttaatttggatttatggtggtggttttcaaacaggtacatcatcattacatgtttatgatggtaaatttttagcaagagttgaaagagttattgttgtttcaatgaattatagagttggtgcattaggttttttagcattaccaggtaatccagaagcaccaggtaatatgggtttatttgatcaacaattagcattacaatgggttcaaaaaaatattgcagcatttggtggtaatccaaaatcagttacattatttggtgaatcagcaggtgcagcatcagtttcattacatttattatcaccaggttcacattcattatttacaagagcaattttacaatcaggttcatttaatgcaccatgggcagttacatcattatatgaagcaagaaatagaacattaaatttagcaaaattaacaggttgttcaagagaaaatgaaacagaaattattaaatgtttaagaaataaagatccacaagaaattttattaaatgaagcatttgttgttccatatggtacaccattatcagttaattttggtccaacagttgatggtgattttttaacagatatgccagatattttattagaattaggtcaatttaaaaaaacacaaattttagttggtgttaataaagatgaaggtacagcatttttagtttatggtgcaccaggtttttcaaaagataataattcaattattacaagaaaagaatttcaagaaggtttaaaaattttttttccaggtgtttcagaatttggtaaagaatcaattttatttcattatacagattgggttgatgatcaaagaccagaaaattatagagaagcattaggtgatgttgttggtgattataattttatttgtccagcattagaatttacaaaaaaattttcagaatggggtaataatgcatttttttattattttgaacatagatcatcaaaattaccatggccagaatggatgggtgttatgcatggttatgaaattgaatttgtttttggtttaccattagaaagaagagataattatacaaaagcagaagaaattttatcaagatcaattgttaaaagatgggcaaattttgcaaaatatggtaatccaaatgaaacacaaaataattcaacatcatggccagtttttaaatcaacagaacaaaaatatttaacattaaatacagaatcaacaagaattatgacaaaattaagagcacaacaatgtagattttggacatcattttttccaaaagttttagaaatgacaggtaatattgatgaagcagaatgggaatggaaagcaggttttcatagatggaataattatatgatggattggaaaaatcaatttaatgattatacatcaaaaaaagaatcatgtgttggttta

>BCHEia

atgagaatgcaattgttgttgttgattgcattgtcattggcattggtgacaaattcagaagatgatattattattgcaacaaaaaatggtaaagtgagaggtatgaatttgacagtgtttggtggtacagtgacagcatttttgggtattccatatgcacaaccaccattgggtagattgagatttaaaaaaccacaatcattgacaaaatggtcagatatttggaatgcaacaaaatatgcaaattcatgttgtcaaaatattgatcaatcatttccaggttttcatggttcagaaatgtggaatccaaatacagatttgtcagaagattgtttgtatttgaatgtgtggattccagcaccaaaaccaaaaaatgcaacagtgttgatttggatttatggtggtggttttcaaacaggtacatcatcattgcatgtgtatgatggtaaatttttggcaagagtggaaagagtgattgtggtgtcaatgaattatagagtgggtgcattgggttttttggcattgccaggtaatccagaagcaccaggtaatatgggtttgtttgatcaacaattggcattgcaatgggtgcaaaaaaatattgcagcatttggtggtaatccaaaatcagtgacattgtttggtgaatcagcaggtgcagcatcagtgtcattgcatttgttgtcaccaggttcacattcattgtttacaagagcaattttgcaatcaggttcatttaatgcaccatgggcagtgacatcattgtatgaagcaagaaatagaacattgaatttggcaaaattgacaggttgttcaagagaaaatgaaacagaaattattaaatgtttgagaaataaagatccacaagaaattttgttgaatgaagcatttgtggtgccatatggtacaccattgtcagtgaattttggtccaacagtggatggtgattttttgacagatatgccagatattttgttggaattgggtcaatttaaaaaaacacaaattttggtgggtgtgaataaagatgaaggtacagcatttttggtgtatggtgcaccaggtttttcaaaagataataattcaattattacaagaaaagaatttcaagaaggtttgaaaattttttttccaggtgtgtcagaatttggtaaagaatcaattttgtttcattatacagattgggtggatgatcaaagaccagaaaattatagagaagcattgggtgatgtggtgggtgattataattttatttgtccagcattggaatttacaaaaaaattttcagaatggggtaataatgcatttttttattattttgaacatagatcatcaaaattgccatggccagaatggatgggtgtgatgcatggttatgaaattgaatttgtgtttggtttgccattggaaagaagagataattatacaaaagcagaagaaattttgtcaagatcaattgtgaaaagatgggcaaattttgcaaaatatggtaatccaaatgaaacacaaaataattcaacatcatggccagtgtttaaatcaacagaacaaaaatatttgacattgaatacagaatcaacaagaattatgacaaaattgagagcacaacaatgtagattttggacatcattttttccaaaagtgttggaaatgacaggtaatattgatgaagcagaatgggaatggaaagcaggttttcatagatggaataattatatgatggattggaaaaatcaatttaatgattatacatcaaaaaaagaatcatgtgtgggtttg

>BCHEec

atgcgtatgcagctgctgctgctgattgcgctgagcctggcgctggtgaccaacagcgaagatgatattattattgcgaccaaaaacggcaaagtgcgtggcatgaacctgaccgtgtttggcggcaccgtgaccgcgtttctgggcattccgtatgcgcagccgccgctgggccgtctgcgttttaaaaaaccgcagagcctgaccaaatggagcgatatttggaacgcgaccaaatatgcgaacagctgctgccagaacattgatcagagctttccgggctttcatggcagcgaaatgtggaacccgaacaccgatctgagcgaagattgcctgtatctgaacgtgtggattccggcgccgaaaccgaaaaacgcgaccgtgctgatttggatttatggcggcggctttcagaccggcaccagcagcctgcatgtgtatgatggcaaatttctggcgcgtgtggaacgtgtgattgtggtgagcatgaactatcgtgtgggcgcgctgggctttctggcgctgccgggcaacccggaagcgccgggcaacatgggcctgtttgatcagcagctggcgctgcagtgggtgcagaaaaacattgcggcgtttggcggcaacccgaaaagcgtgaccctgtttggcgaaagcgcgggcgcggcgagcgtgagcctgcatctgctgagcccgggcagccatagcctgtttacccgtgcgattctgcagagcggcagctttaacgcgccgtgggcggtgaccagcctgtatgaagcgcgtaaccgtaccctgaacctggcgaaactgaccggctgcagccgtgaaaacgaaaccgaaattattaaatgcctgcgtaacaaagatccgcaggaaattctgctgaacgaagcgtttgtggtgccgtatggcaccccgctgagcgtgaactttggcccgaccgtggatggcgattttctgaccgatatgccggatattctgctggaactgggccagtttaaaaaaacccagattctggtgggcgtgaacaaagatgaaggcaccgcgtttctggtgtatggcgcgccgggctttagcaaagataacaacagcattattacccgtaaagaatttcaggaaggcctgaaaattttttttccgggcgtgagcgaatttggcaaagaaagcattctgtttcattataccgattgggtggatgatcagcgtccggaaaactatcgtgaagcgctgggcgatgtggtgggcgattataactttatttgcccggcgctggaatttaccaaaaaatttagcgaatggggcaacaacgcgtttttttattattttgaacatcgtagcagcaaactgccgtggccggaatggatgggcgtgatgcatggctatgaaattgaatttgtgtttggcctgccgctggaacgtcgtgataactataccaaagcggaagaaattctgagccgtagcattgtgaaacgttgggcgaactttgcgaaatatggcaacccgaacgaaacccagaacaacagcaccagctggccggtgtttaaaagcaccgaacagaaatatctgaccctgaacaccgaaagcacccgtattatgaccaaactgcgtgcgcagcagtgccgtttttggaccagcttttttccgaaagtgctggaaatgaccggcaacattgatgaagcggaatgggaatggaaagcgggctttcatcgttggaacaactatatgatggattggaaaaaccagtttaacgattataccagcaaaaaagaaagctgcgtgggcctg

>BCHEeb

atgagaatgcaacttcttcttcttattgcactttcacttgcacttgttacaaattcagaagatgatattattattgcaacaaaaaatggaaaagttagaggaatgaatcttacagttttcggaggaacagttacagcattccttggaattccatatgcacaaccaccacttggaagacttagattcaaaaaaccacaatcacttacaaaatggtcagatatttggaatgcaacaaaatatgcaaattcatgttgtcaaaatattgatcaatcattcccaggattccatggatcagaaatgtggaatccaaatacagatctttcagaagattgtctttatcttaatgtttggattccagcaccaaaaccaaaaaatgcaacagttcttatttggatttatggaggaggattccaaacaggaacatcatcacttcatgtttatgatggaaaattccttgcaagagttgaaagagttattgttgtttcaatgaattatagagttggagcacttggattccttgcacttccaggaaatccagaagcaccaggaaatatgggacttttcgatcaacaacttgcacttcaatgggttcaaaaaaatattgcagcattcggaggaaatccaaaatcagttacacttttcggagaatcagcaggagcagcatcagtttcacttcatcttctttcaccaggatcacattcacttttcacaagagcaattcttcaatcaggatcattcaatgcaccatgggcagttacatcactttatgaagcaagaaatagaacacttaatcttgcaaaacttacaggatgttcaagagaaaatgaaacagaaattattaaatgtcttagaaataaagatccacaagaaattcttcttaatgaagcattcgttgttccatatggaacaccactttcagttaatttcggaccaacagttgatggagatttccttacagatatgccagatattcttcttgaacttggacaattcaaaaaaacacaaattcttgttggagttaataaagatgaaggaacagcattccttgtttatggagcaccaggattctcaaaagataataattcaattattacaagaaaagaattccaagaaggacttaaaattttcttcccaggagtttcagaattcggaaaagaatcaattcttttccattatacagattgggttgatgatcaaagaccagaaaattatagagaagcacttggagatgttgttggagattataatttcatttgtccagcacttgaattcacaaaaaaattctcagaatggggaaataatgcattcttctattatttcgaacatagatcatcaaaacttccatggccagaatggatgggagttatgcatggatatgaaattgaattcgttttcggacttccacttgaaagaagagataattatacaaaagcagaagaaattctttcaagatcaattgttaaaagatgggcaaatttcgcaaaatatggaaatccaaatgaaacacaaaataattcaacatcatggccagttttcaaatcaacagaacaaaaatatcttacacttaatacagaatcaacaagaattatgacaaaacttagagcacaacaatgtagattctggacatcattcttcccaaaagttcttgaaatgacaggaaatattgatgaagcagaatgggaatggaaagcaggattccatagatggaataattatatgatggattggaaaaatcaattcaatgattatacatcaaaaaaagaatcatgtgttggactt

>BCHE39

atgagaatgcagctgctgctgctgatcgctctgtcactggctctggtgaccaactcagaggatgatatcatcatcgctaccaaaaacggaaaagtgagaggaatgaacctgaccgtgttcggaggaaccgtgaccgctttcctgggaatcccctatgctcagccccccctgggaagactgagattcaaaaaaccccagtcactgaccaaatggtcagatatctggaacgctaccaaatatgctaactcatgctgccagaacatcgatcagtcattccccggattccatggatcagagatgtggaaccccaacaccgatctgtcagaggattgcctgtatctgaacgtgtggatccccgctcccaaacccaaaaacgctaccgtgctgatctggatctatggaggaggattccagaccggaacctcatcactgcatgtgtatgatggaaaattcctggctagagtggagagagtgatcgtggtgtcaatgaactatagagtgggagctctgggattcctggctctgcccggaaaccccgaggctcccggaaacatgggactgttcgatcagcagctggctctgcagtgggtgcagaaaaacatcgctgctttcggaggaaaccccaaatcagtgaccctgttcggagagtcagctggagctgcttcagtgtcactgcatctgctgtcacccggatcacattcactgttcaccagagctatcctgcagtcaggatcattcaacgctccctgggctgtgacctcactgtatgaggctagaaacagaaccctgaacctggctaaactgaccggatgctcaagagagaacgagaccgagatcatcaaatgcctgagaaacaaagatccccaggagatcctgctgaacgaggctttcgtggtgccctatggaacccccctgtcagtgaacttcggacccaccgtggatggagatttcctgaccgatatgcccgatatcctgctggagctgggacagttcaaaaaaacccagatcctggtgggagtgaacaaagatgagggaaccgctttcctggtgtatggagctcccggattctcaaaagataacaactcaatcatcaccagaaaagagttccaggagggactgaaaatcttcttccccggagtgtcagagttcggaaaagagtcaatcctgttccattataccgattgggtggatgatcagagacccgagaactatagagaggctctgggagatgtggtgggagattataacttcatctgccccgctctggagttcaccaaaaaattctcagagtggggaaacaacgctttcttctattatttcgagcatagatcatcaaaactgccctggcccgagtggatgggagtgatgcatggatatgagatcgagttcgtgttcggactgcccctggagagaagagataactataccaaagctgaggagatcctgtcaagatcaatcgtgaaaagatgggctaacttcgctaaatatggaaaccccaacgagacccagaacaactcaacctcatggcccgtgttcaaatcaaccgagcagaaatatctgaccctgaacaccgagtcaaccagaatcatgaccaaactgagagctcagcagtgcagattctggacctcattcttccccaaagtgctggagatgaccggaaacatcgatgaggctgagtgggagtggaaagctggattccatagatggaacaactatatgatggattggaaaaaccagttcaacgattatacctcaaaaaaagagtcatgcgtgggactg

>BCHE10a

atgcgaatgcagctgctgctgctgatcgcactgagcctggcactggtgaccaacagcgaggacgacatcatcatcgcaaccaagaacggaaaggtgcgaggaatgaacctgaccgtgttcggaggaaccgtgaccgcattcctgggaatcccatacgcacagccaccactgggacgactgcgattcaagaagccacagagcctgaccaagtggagcgacatctggaacgcaaccaagtacgcaaacagctgttgtcagaacatcgaccagagcttcccaggattccatggaagcgagatgtggaacccaaacaccgacctgagcgaggactgtctgtacctgaacgtgtggatcccagcaccaaagccaaagaacgcaaccgtgctgatctggatctacggaggaggattccagaccggaaccagcagcctgcatgtgtacgacggaaagttcctggcacgagtggagcgagtgatcgtggtgagcatgaactaccgagtgggagcactgggattcctggcactgccaggaaacccagaggcaccaggaaacatgggactgttcgaccagcagctggcactgcagtgggtgcagaagaacatcgcagcattcggaggaaacccaaagagcgtgaccctgttcggagagagcgcaggagcagcaagcgtgagcctgcatctgctgagcccaggaagccatagcctgttcacccgagcaatcctgcagagcggaagcttcaacgcaccatgggcagtgaccagcctgtacgaggcacgaaaccgaaccctgaacctggcaaagctgaccggatgtagccgagagaacgagaccgagatcatcaagtgtctgcgaaacaaggacccacaggagatcctgctgaacgaggcattcgtggtgccatacggaaccccactgagcgtgaacttcggaccaaccgtggacggagacttcctgaccgacatgccagacatcctgctggagctgggacagttcaagaagacccagatcctggtgggagtgaacaaggacgagggaaccgcattcctggtgtacggagcaccaggattcagcaaggacaacaacagcatcatcacccgaaaggagttccaggagggactgaagatcttcttcccaggagtgagcgagttcggaaaggagagcatcctgttccattacaccgactgggtggacgaccagcgaccagagaactaccgagaggcactgggagacgtggtgggagactacaacttcatctgtccagcactggagttcaccaagaagttcagcgagtggggaaacaacgcattcttctactacttcgagcatcgaagcagcaagctgccatggccagagtggatgggagtgatgcatggatacgagatcgagttcgtgttcggactgccactggagcgacgagacaactacaccaaggcagaggagatcctgagccgaagcatcgtgaagcgatgggcaaacttcgcaaagtacggaaacccaaacgagacccagaacaacagcaccagctggccagtgttcaagagcaccgagcagaagtacctgaccctgaacaccgagagcacccgaatcatgaccaagctgcgagcacagcagtgtcgattctggaccagcttcttcccaaaggtgctggagatgaccggaaacatcgacgaggcagagtgggagtggaaggcaggattccatcgatggaacaactacatgatggactggaagaaccagttcaacgactacaccagcaagaaggagagctgtgtgggactg

>BCHE11

atgcggatgcagctgctgctgctgattgctctgagcctggctctggtgaccaacagcgaagacgacattattattgctaccaagaacggcaaggtgcggggcatgaacctgaccgtgtttggcggcaccgtgaccgcttttctgggcattccctatgctcagccccccctgggccggctgcggtttaagaagccccagagcctgaccaagtggagcgacatttggaacgctaccaagtatgctaacagctgttgtcagaacattgaccagagctttcccggctttcatggcagcgaaatgtggaaccccaacaccgacctgagcgaagactgtctgtatctgaacgtgtggattcccgctcccaagcccaagaacgctaccgtgctgatttggatttatggcggcggctttcagaccggcaccagcagcctgcatgtgtatgacggcaagtttctggctcgggtggaacgggtgattgtggtgagcatgaactatcgggtgggcgctctgggctttctggctctgcccggcaaccccgaagctcccggcaacatgggcctgtttgaccagcagctggctctgcagtgggtgcagaagaacattgctgcttttggcggcaaccccaagagcgtgaccctgtttggcgaaagcgctggcgctgctagcgtgagcctgcatctgctgagccccggcagccatagcctgtttacccgggctattctgcagagcggcagctttaacgctccctgggctgtgaccagcctgtatgaagctcggaaccggaccctgaacctggctaagctgaccggctgtagccgggaaaacgaaaccgaaattattaagtgtctgcggaacaaggacccccaggaaattctgctgaacgaagcttttgtggtgccctatggcacccccctgagcgtgaactttggccccaccgtggacggcgactttctgaccgacatgcccgacattctgctggaactgggccagtttaagaagacccagattctggtgggcgtgaacaaggacgaaggcaccgcttttctggtgtatggcgctcccggctttagcaaggacaacaacagcattattacccggaaggaatttcaggaaggcctgaagattttttttcccggcgtgagcgaatttggcaaggaaagcattctgtttcattataccgactgggtggacgaccagcggcccgaaaactatcgggaagctctgggcgacgtggtgggcgactataactttatttgtcccgctctggaatttaccaagaagtttagcgaatggggcaacaacgcttttttttattattttgaacatcggagcagcaagctgccctggcccgaatggatgggcgtgatgcatggctatgaaattgaatttgtgtttggcctgcccctggaacggcgggacaactataccaaggctgaagaaattctgagccggagcattgtgaagcggtgggctaactttgctaagtatggcaaccccaacgaaacccagaacaacagcaccagctggcccgtgtttaagagcaccgaacagaagtatctgaccctgaacaccgaaagcacccggattatgaccaagctgcgggctcagcagtgtcggttttggaccagcttttttcccaaggtgctggaaatgaccggcaacattgacgaagctgaatgggaatggaaggctggctttcatcggtggaacaactatatgatggactggaagaaccagtttaacgactataccagcaagaaggaaagctgtgtgggcctg

>BCHE201

atgcggatgcagctgctgctgctgatcgccctgagcctggccctggtgaccaacagcgaggatgatatcatcatcgccaccaagaacggaaaggtgcggggaatgaacctgaccgtgttcggaggaaccgtgaccgccttcctgggaatcccatacgcccagccaccactgggacggctgcggttcaagaagccacagagcctgaccaagtggagcgatatctggaacgccaccaagtacgccaacagctgctgccagaacatcgatcagagcttcccaggattccacggaagcgagatgtggaacccaaacaccgatctgagcgaggattgcctgtacctgaacgtgtggatcccagccccaaagccaaagaacgccaccgtgctgatctggatctacggaggaggattccagaccggaaccagcagcctgcacgtgtacgatggaaagttcctggcccgggtggagcgggtgatcgtggtgagcatgaactaccgggtgggagccctgggattcctggccctgccaggaaacccagaggccccaggaaacatgggactgttcgatcagcagctggccctgcagtgggtgcagaagaacatcgccgccttcggaggaaacccaaagagcgtgaccctgttcggagagagcgccggagccgccagcgtgagcctgcacctgctgagcccaggaagccacagcctgttcacccgggccatcctgcagagcggaagcttcaacgccccatgggccgtgaccagcctgtacgaggcccggaaccggaccctgaacctggccaagctgaccggatgcagccgggagaacgagaccgagatcatcaagtgcctgcggaacaaggatccacaggagatcctgctgaacgaggccttcgtggtgccatacggaaccccactgagcgtgaacttcggaccaaccgtggatggagatttcctgaccgatatgccagatatcctgctggagctgggacagttcaagaagacccagatcctggtgggagtgaacaaggatgagggaaccgccttcctggtgtacggagccccaggattcagcaaggataacaacagcatcatcacccggaaggagttccaggagggactgaagatcttcttcccaggagtgagcgagttcggaaaggagagcatcctgttccactacaccgattgggtggatgatcagcggccagagaactaccgggaggccctgggagatgtggtgggagattacaacttcatctgcccagccctggagttcaccaagaagttcagcgagtggggaaacaacgccttcttctactacttcgagcaccggagcagcaagctgccatggccagagtggatgggagtgatgcacggatacgagatcgagttcgtgttcggactgccactggagcggcgggataactacaccaaggccgaggagatcctgagccggagcatcgtgaagcggtgggccaacttcgccaagtacggaaacccaaacgagacccagaacaacagcaccagctggccagtgttcaagagcaccgagcagaagtacctgaccctgaacaccgagagcacccggatcatgaccaagctgcgggcccagcagtgccggttctggaccagcttcttcccaaaggtgctggagatgaccggaaacatcgatgaggccgagtgggagtggaaggccggattccaccggtggaacaactacatgatggattggaagaaccagttcaacgattacaccagcaagaaggagagctgcgtgggactg

>BCHE30

atgagaatgcagcttcttcttcttatcgctcttagccttgctcttgtgacaaacagcgaagatgatatcatcatcgctacaaagaacgggaaggtgagagggatgaaccttacagtgtttggggggacagtgacagcttttcttgggatcccttatgctcagcctcctcttgggagacttagatttaagaagcctcagagccttacaaagtggagcgatatctggaacgctacaaagtatgctaacagctgttgtcagaacatcgatcagagctttcctgggtttcacgggagcgaaatgtggaaccctaacacagatcttagcgaagattgtctttatcttaacgtgtggatccctgctcctaagcctaagaacgctacagtgcttatctggatctatgggggggggtttcagacagggacaagcagccttcacgtgtatgatgggaagtttcttgctagagtggaaagagtgatcgtggtgagcatgaactatagagtgggggctcttgggtttcttgctcttcctgggaaccctgaagctcctgggaacatggggctttttgatcagcagcttgctcttcagtgggtgcagaagaacatcgctgcttttggggggaaccctaagagcgtgacactttttggggaaagcgctggggctgctagcgtgagccttcaccttcttagccctgggagccacagcctttttacaagagctatccttcagagcgggagctttaacgctccttgggctgtgacaagcctttatgaagctagaaacagaacacttaaccttgctaagcttacagggtgtagcagagaaaacgaaacagaaatcatcaagtgtcttagaaacaaggatcctcaggaaatccttcttaacgaagcttttgtggtgccttatgggacacctcttagcgtgaactttgggcctacagtggatggggattttcttacagatatgcctgatatccttcttgaacttgggcagtttaagaagacacagatccttgtgggggtgaacaaggatgaagggacagcttttcttgtgtatggggctcctgggtttagcaaggataacaacagcatcatcacaagaaaggaatttcaggaagggcttaagatcttttttcctggggtgagcgaatttgggaaggaaagcatcctttttcactatacagattgggtggatgatcagagacctgaaaactatagagaagctcttggggatgtggtgggggattataactttatctgtcctgctcttgaatttacaaagaagtttagcgaatgggggaacaacgcttttttttattattttgaacacagaagcagcaagcttccttggcctgaatggatgggggtgatgcacgggtatgaaatcgaatttgtgtttgggcttcctcttgaaagaagagataactatacaaaggctgaagaaatccttagcagaagcatcgtgaagagatgggctaactttgctaagtatgggaaccctaacgaaacacagaacaacagcacaagctggcctgtgtttaagagcacagaacagaagtatcttacacttaacacagaaagcacaagaatcatgacaaagcttagagctcagcagtgtagattttggacaagcttttttcctaaggtgcttgaaatgacagggaacatcgatgaagctgaatgggaatggaaggctgggtttcacagatggaacaactatatgatggattggaagaaccagtttaacgattatacaagcaagaaggaaagctgtgtggggctt

>PRIMA1

Atgcaactcctgtcttgcattgcactaagtcttgcacttgtcacaaacagtccacagaagagctgcagcaaggtgaccgatagctgccggcacgtgtgccagtgccggccaccaccaccactgccaccaccaccaccaccaccaccaccaccacggctgctgagcgccccagccccaaacagcaccagctgcccaaccgaggagagctggtggagcgga

>LAMELLIPODIN

Atgcaactcctgtcttgcattgcactaagtcttgcacttgtcacaaacagtccacagccaaagatcgtgaccccatacaccgccagccagccaagcccaccactgccaccaccaccaccaccaccaccaccaccaccaccaccaccaccaccaccaccaccaccactgccaagccagagcgccccaagcgccggaagcgccgccccaatgttcgtgaagtacagcaccatcacccggctgcagaacgccagccagcacagcgga

>BCHEfusedtomodifiedIL2signal

Met Arg Met Gln Leu Leu Leu Leu Ile Ala Leu Ser Leu Ala Leu Val Thr Asn Ser Glu Asp Asp Ile Ile Ile Ala Thr Lys Asn Gly Lys Val Arg Gly Met Asn Leu Thr Val Phe Gly Gly Thr Val Thr Ala Phe Leu Gly Ile Pro Tyr Ala Gln Pro Pro Leu Gly Arg Leu Arg Phe Lys Lys Pro Gln Ser Leu Thr Lys Trp Ser Asp Ile Trp Asn Ala Thr Lys Tyr Ala Asn Ser Cys Cys Gln Asn Ile Asp Gln Ser Phe Pro Gly Phe His Gly Ser Glu Met Trp Asn Pro Asn Thr Asp Leu Ser Glu Asp Cys Leu Tyr Leu Asn Val Trp Ile Pro Ala Pro Lys Pro Lys Asn Ala Thr Val Leu Ile Trp Ile Tyr Gly Gly Gly Phe Gln Thr Gly Thr Ser Ser Leu His Val Tyr Asp Gly Lys Phe Leu Ala Arg Val Glu Arg Val Ile Val Val Ser Met Asn Tyr Arg Val Gly Ala Leu Gly Phe Leu Ala Leu Pro Gly Asn Pro Glu Ala Pro Gly Asn Met Gly Leu Phe Asp Gln Gln Leu Ala Leu Gln Trp Val Gln Lys Asn Ile Ala Ala Phe Gly Gly Asn Pro Lys Ser Val Thr Leu Phe Gly Glu Ser Ala Gly Ala Ala Ser Val Ser Leu His Leu Leu Ser Pro Gly Ser His Ser Leu Phe Thr Arg Ala Ile Leu Gln Ser Gly Ser Phe Asn Ala Pro Trp Ala Val Thr Ser Leu Tyr Glu Ala Arg Asn Arg Thr Leu Asn Leu Ala Lys Leu Thr Gly Cys Ser Arg Glu Asn Glu Thr Glu Ile Ile Lys Cys Leu Arg Asn Lys Asp Pro Gln Glu Ile Leu Leu Asn Glu Ala Phe Val Val Pro Tyr Gly Thr Pro Leu Ser Val Asn Phe Gly Pro Thr Val Asp Gly Asp Phe Leu Thr Asp Met Pro Asp Ile Leu Leu Glu Leu Gly Gln Phe Lys Lys Thr Gln Ile Leu Val Gly Val Asn Lys Asp Glu Gly Thr Ala Phe Leu Val Tyr Gly Ala Pro Gly Phe Ser Lys Asp Asn Asn Ser Ile Ile Thr Arg Lys Glu Phe Gln Glu Gly Leu Lys Ile Phe Phe Pro Gly Val Ser Glu Phe Gly Lys Glu Ser Ile Leu Phe His Tyr Thr Asp Trp Val Asp Asp Gln Arg Pro Glu Asn Tyr Arg Glu Ala Leu Gly Asp Val Val Gly Asp Tyr Asn Phe Ile Cys Pro Ala Leu Glu Phe Thr Lys Lys Phe Ser Glu Trp Gly Asn Asn Ala Phe Phe Tyr Tyr Phe Glu His Arg Ser Ser Lys Leu Pro Trp Pro Glu Trp Met Gly Val Met His Gly Tyr Glu Ile Glu Phe Val Phe Gly Leu Pro Leu Glu Arg Arg Asp Asn Tyr Thr Lys Ala Glu Glu Ile Leu Ser Arg Ser Ile Val Lys Arg Trp Ala Asn Phe Ala Lys Tyr Gly Asn Pro Asn Glu Thr Gln Asn Asn Ser Thr Ser Trp Pro Val Phe Lys Ser Thr Glu Gln Lys Tyr Leu Thr Leu Asn Thr Glu Ser Thr Arg Ile Met Thr Lys Leu Arg Ala Gln Gln Cys Arg Phe Trp Thr Ser Phe Phe Pro Lys Val Leu Glu Met Thr Gly Asn Ile Asp Glu Ala Glu Trp Glu Trp Lys Ala Gly Phe His Arg Trp Asn Asn Tyr Met Met Asp Trp Lys Asn Gln Phe Asn Asp Tyr Thr Ser Lys Lys Glu Ser Cys Val Gly Leu

>PRIMA1prolinerichfragmentfusedtoIL2signal

Met Gln Leu Leu Ser Cys Ile Ala Leu Ser Leu Ala Leu Val Thr Asn Ser Pro Gln Lys Ser Cys Ser Lys Val Thr Asp Ser Cys Arg His Val Cys Gln Cys Arg Pro Pro Pro Pro Leu Pro Pro Pro Pro Pro Pro Pro Pro Pro Pro Arg Leu Leu Ser Ala Pro Ala Pro Asn Ser Thr Ser Cys Pro Thr Glu Glu Ser Trp Trp Ser Gly

>LAMELLIPODINprolinerichfragmentfusedtoIL2signal

Met Gln Leu Leu Ser Cys Ile Ala Leu Ser Leu Ala Leu Val Thr Asn Ser Pro Gln Pro Lys Ile Val Thr Pro Tyr Thr Ala Ser Gln Pro Ser Pro Pro Leu Pro Pro Pro Pro Pro Pro Pro Pro Pro Pro Pro Pro Pro Pro Pro Pro Pro Pro Pro Pro Leu Pro Ser Gln Ser Ala Pro Ser Ala Gly Ser Ala Ala Pro Met Phe Val Lys Tyr Ser Thr Ile Thr Arg Leu Gln Asn Ala Ser Gln His Ser Gly
