# Supplementary material for: Adeno-associated virus-mediated expression of human butyrylcholinesterase to treat organophosphate poisoning
Source: PLoS One. 2019 Nov 25;14(11):e0225188. doi: 10.1371/journal.pone.0225188 (PMC6876934; doi:10.1371/journal.pone.0225188)
Supplement: S1 File — (DOCX) [file pone.0225188.s002.docx]

**Supplemental Material: Abbreviations**

| AAV | Adeno-Associated Virus |
| --- | --- |
| AChE | Acetylcholinesterase |
| BChE | Butyrylcholinesterase |
| BL | Baseline |
| CbA | Chicken β-actin |
| CB7 | Hybrid C4 and Chicken β Actin Promoter |
| CMV | Cytomegalovirus Promoter |
| EMCV | Encephalomyocarditis Virus IRES |
| ES1 | Carboxylesterase 1 |
| GC | Genome Copies |
| GT | Gene Therapy |
| hBChE | Human Butyrylcholinesterase |
| hIL2 | Human Interleukin 2 Leader Peptide |
| IM | Intramuscular |
| IRES | Internal Ribosome Entry Site |
| ITR | Inverted Terminal Repeats |
| KO | Knock out |
| 2xLD_50_ | 2 x Lethal Dose 50 |
| LPDN | Lamellipodin |
| OPs | Organophosphates |
| pp | Polyproline Rich Peptide |
| PRIMA | Proline-Rich Membrane Anchor |
| RAG | Recombination Activating Gene |
| rBG | Rabbit β-Globin Polyadenylation Sequence |
| SD | Standard Deviation |
| SV40 | Simian Virus 40 Polyadenylation Sequence |
| UbC | Ubiquitin C Promoter |
| VX | Venomous agent X |
