# Supplementary material for: Adeno-associated virus-mediated expression of human butyrylcholinesterase to treat organophosphate poisoning
Source: PLoS One. 2019 Nov 25;14(11):e0225188. doi: 10.1371/journal.pone.0225188 (PMC6876934; doi:10.1371/journal.pone.0225188)
Supplement: S1 Fig — Male BChE KO mice (n = 3/group) were injected IM with 1011 GC/mouse of AAV-BChE vectors as shown. Data is shown as the average of all mice (n) ± SD. (DOCX) [file pone.0225188.s003.docx]

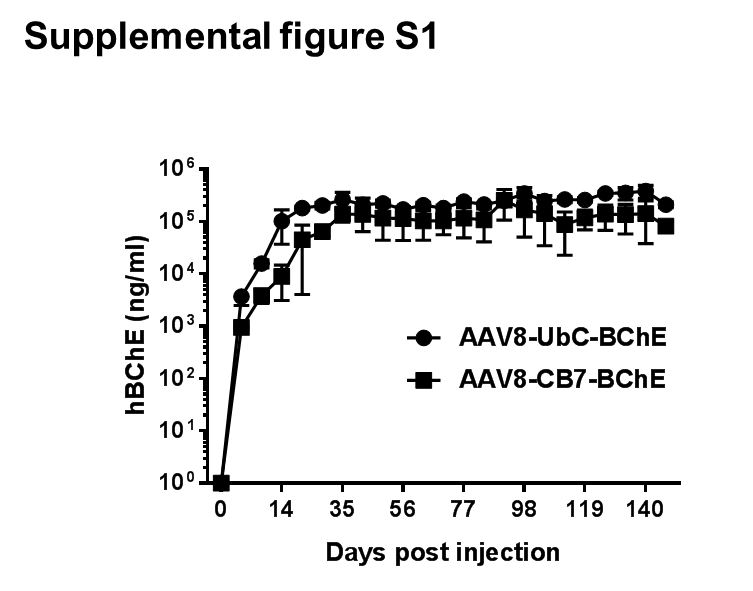


**Days post injection into BChE KO mice**

**Figure S1.** **Long-term expression of hBChE (ng/ml) in the serum.** Male BChE KO mice (n=3/group) were injected IM with 10^11^ GC/mouse of AAV-BChE vectors as shown. Data is shown as the average of all mice (n) ± SD.
