# Supplementary material for: Adeno-associated virus-mediated expression of human butyrylcholinesterase to treat organophosphate poisoning
Source: PLoS One. 2019 Nov 25;14(11):e0225188. doi: 10.1371/journal.pone.0225188 (PMC6876934; doi:10.1371/journal.pone.0225188)
Supplement: S2 Fig — Male (blue squares) and female (red circles) ES1 KO mice (n = 5/group) were injected IM with 1012 GC/mouse of AAV9-C4/UbC-BChE or AAV9-CB7-BChE vectors as shown. Data from an individual animal is shown. Brief methodology: Purified hBChE protein (1μg/ml, diluted in PBS) was used to coat high binding 96-well plates overnight at 4°C. The next day, plates were washed 3X with PBST and blocked with 1% BSA in PBS. Later on, 50 μl of different serum dilutions were added to all wells and incubated for 1 hr at room temperature. Plates were then washed 3X with PBST, and treated with primary antibodies (anti-mouse IgG-Biotin, 0.2ug/ml, catalogue# ab6788, 3 μl in 30 ml PBST) diluted in PBST as per the manufacturer’s recommendation; for 1 hr at room temperature. Plates were washed again, treated with secondary antibody (Biotin-HRP) and developed for reading optical density (OD) at 450 nm. (DOCX) [file pone.0225188.s004.docx]

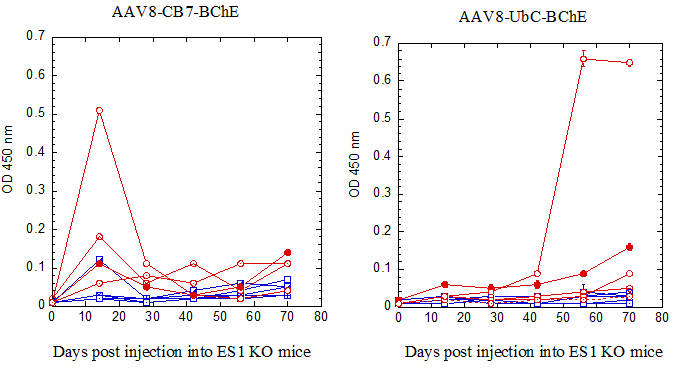


**Figure S2. Expression of anti-hBChE IgG antibodies in ES1 KO mice.** Male (Blue squares) and female (Red circles) ES1 KO mice (n=5/group) were injected IM with 10^12^ GC/mouse of AAV9-C4/UbC-BChE or AAV9-CB7-BChE vectors as shown. Data of individual animal (Serum dilution 1: 200) is shown.

Brief methodology: Purified hBChE protein (1µg/ml, diluted in PBS) was used to coat high binding 96-well plates O/N at 4°C. The next day, plates were washed 3X with PBST and blocked with 1% BSA in PBS. Later on, 50 µl of different serum dilutions were added to all wells and incubated at 1hr at room temperature. Plates were then washed 3X with PBST, and treated with primary antibodies (anti-Mouse IgG-Biotin, 0.2ug/ml, catalogue# ab6788, 3 µl in 30 ml PBST) diluted in PBST as per the manufacturer’s recommendation; for 1 hr. at room temperature. Plates were washed again, treated with secondary antibody (Biotin-HRP) and developed for reading optical density (OD) at 450 nm.
