# Supplementary material for: Adeno-associated virus-mediated expression of human butyrylcholinesterase to treat organophosphate poisoning
Source: PLoS One. 2019 Nov 25;14(11):e0225188. doi: 10.1371/journal.pone.0225188 (PMC6876934; doi:10.1371/journal.pone.0225188)
Supplement: S3 Fig — In-gel activity assay of hBChE expressed by AAV8-CB7-BChE vector in RAG KO mice serum. BL = Baseline. (DOCX) [file pone.0225188.s005.docx]

**
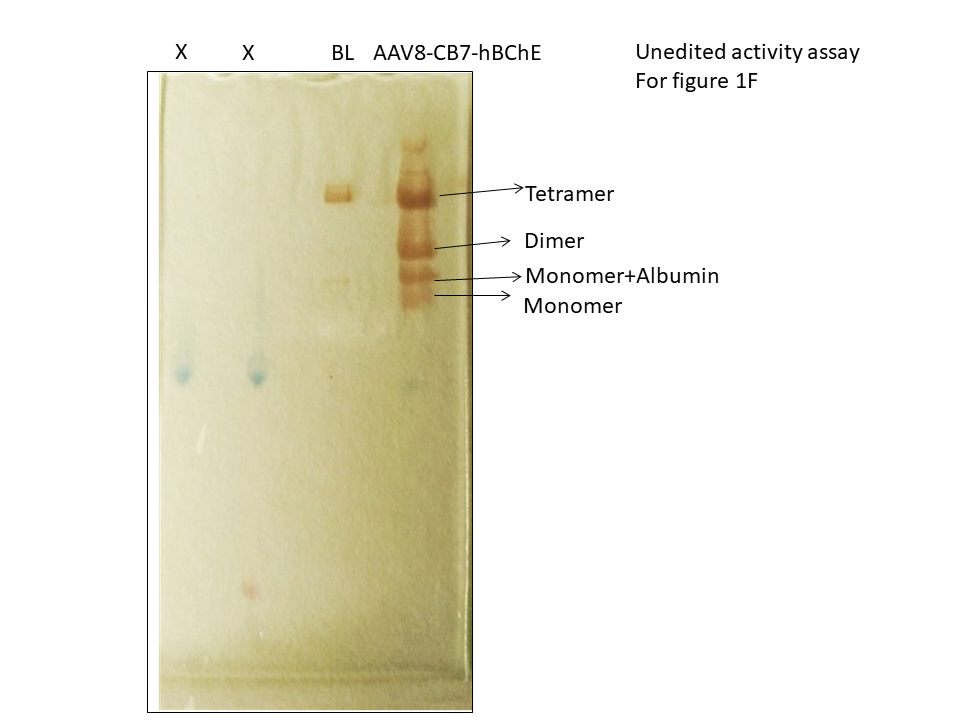
**

**Figure S3**. **Raw data for in-gel activity assay (for Figure 1F).** In-gel activity assay of hBChE expressed by AAV8-CB7-BChE vector in RAG KO mice serum. BL = Baseline.
