# Supplementary material for: Adeno-associated virus-mediated expression of human butyrylcholinesterase to treat organophosphate poisoning
Source: PLoS One. 2019 Nov 25;14(11):e0225188. doi: 10.1371/journal.pone.0225188 (PMC6876934; doi:10.1371/journal.pone.0225188)
Supplement: S4 Fig — Raw data for Western blot of hBChE expressed by AAV8-CB7-BChE vector in RAG KO mice serum. (DOCX) [file pone.0225188.s006.docx]

**
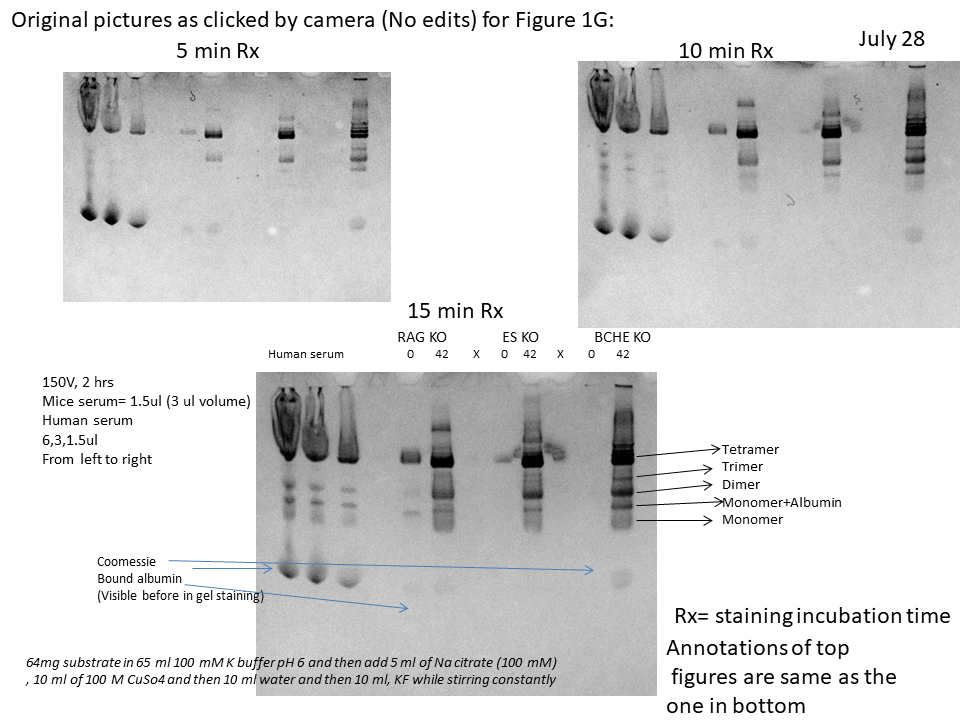
**

**Figure S4**. **Raw data for Western blot (for Figure 1G).** Raw data for Western blot of hBChE expressed by AAV8-CB7-BChE vector in RAG KO mice serum
