# Supplementary material for: Adeno-associated virus-mediated expression of human butyrylcholinesterase to treat organophosphate poisoning
Source: PLoS One. 2019 Nov 25;14(11):e0225188. doi: 10.1371/journal.pone.0225188 (PMC6876934; doi:10.1371/journal.pone.0225188)
Supplement: S5 Fig — Male and female ES1 KO mice (n = 5/group) were injected IM with 1012 GC/mouse of AAV9-C4/UbC-BChE vector as shown. Data is shown as the average of all mice (n) ± SD. The post-challenge survival is shown in the main article (Fig 2H). (DOCX) [file pone.0225188.s007.docx]

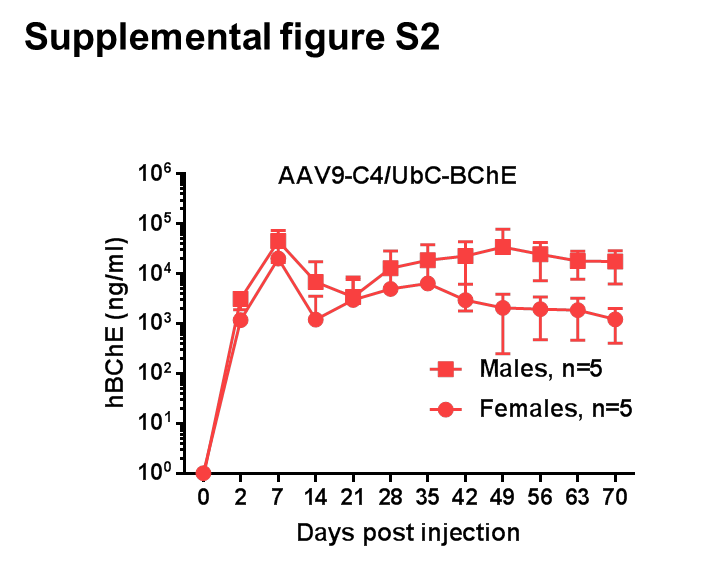


**Days post injection into ES1 KO mice**

**Figure S5. Expression of hBChE (ng/ml) in the plasma of ES1 KO mice.** Male and female ES1 KO mice (n=5/group) were injected IM with 10^12^ GC/mouse of AAV9-C4/UbC-BChE vector as shown. Data is shown as the average of all mice (n) ± SD. The post-challenge survival is shown in the main article (Fig. 2h).
