# Supplementary material for: Adeno-associated virus-mediated expression of human butyrylcholinesterase to treat organophosphate poisoning
Source: PLoS One. 2019 Nov 25;14(11):e0225188. doi: 10.1371/journal.pone.0225188 (PMC6876934; doi:10.1371/journal.pone.0225188)
Supplement: S6 Fig — Male and female ES1 KO mice (n = 5/group) were injected IM with 1012 GC/mouse of different AAV-BChE vectors as shown. Approximately 2.2 μM of hBChE is required to neutralize 2×LD50 VX. Data is shown as the average of all mice (n) ± SD. (DOCX) [file pone.0225188.s008.docx]

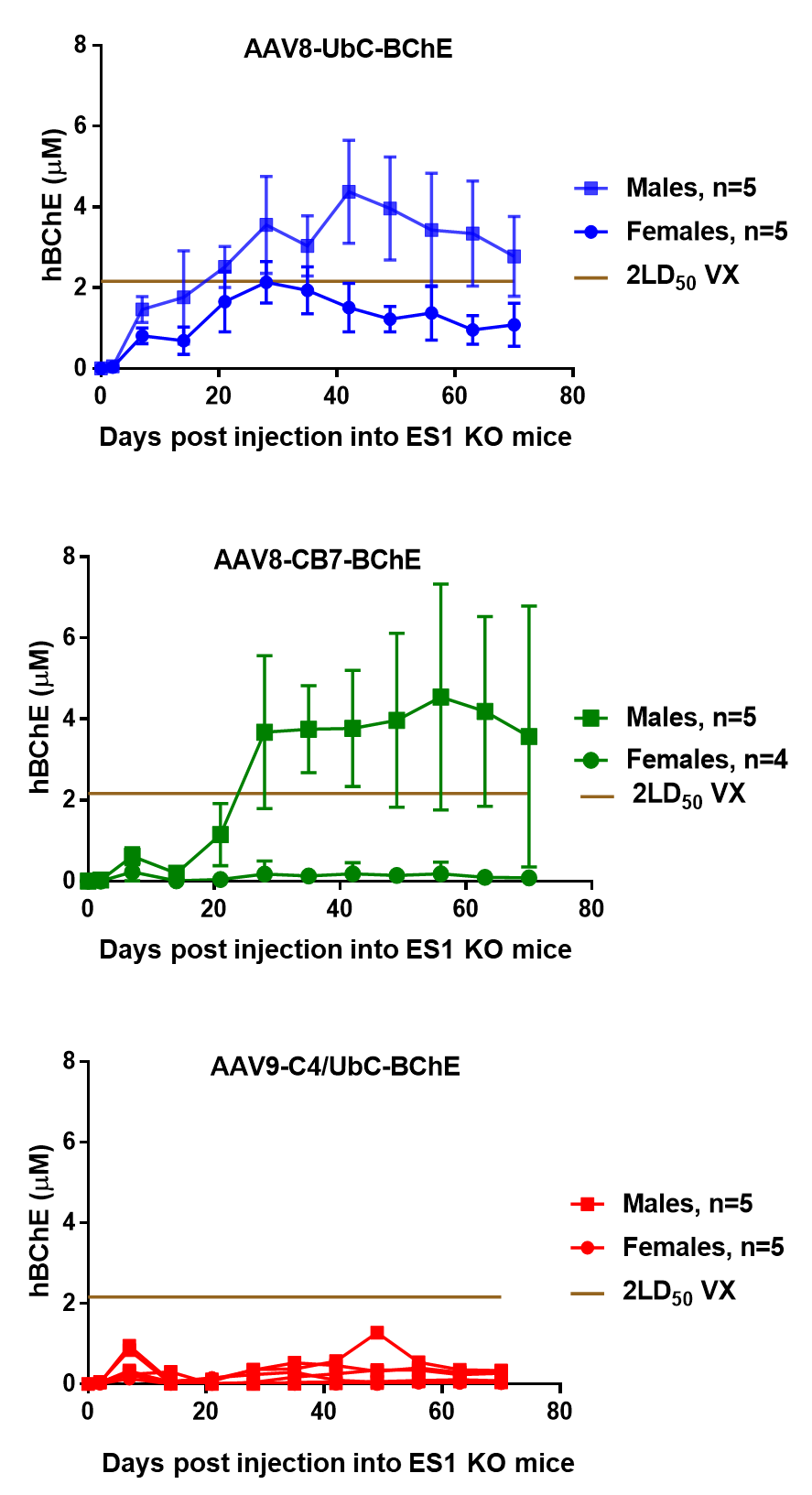


**Figure S6. Expression of hBChE (µM) in the plasma of ES1 KO mice.** Male and female ES1 KO mice (n=5/group) were injected IM with 10^12^ GC/mouse of different AAV-BChE vectors as shown. Approximately 2.2 µM of hBChE is required to neutralize 2×LD_50_ VX. Data is shown as the average of all mice (n) ± SD
